# Eligibility Traces and Plasticity on Behavioral Time Scales: Experimental Support of NeoHebbian Three-Factor Learning Rules

**DOI:** 10.3389/fncir.2018.00053

**Published:** 2018-07-31

**Authors:** Wulfram Gerstner, Marco Lehmann, Vasiliki Liakoni, Dane Corneil, Johanni Brea

**Affiliations:** School of Computer Science and School of Life Sciences, École Polytechnique Fédérale de Lausanne, Lausanne, Switzerland

**Keywords:** eligibility trace, hebb rule, reinforcement learning, neuromodulators, surprise, synaptic tagging, synaptic plasticity, behavioral learning

## Abstract

Most elementary behaviors such as moving the arm to grasp an object or walking into the next room to explore a museum evolve on the time scale of seconds; in contrast, neuronal action potentials occur on the time scale of a few milliseconds. Learning rules of the brain must therefore bridge the gap between these two different time scales. Modern theories of synaptic plasticity have postulated that the co-activation of pre- and postsynaptic neurons sets a flag at the synapse, called an eligibility trace, that leads to a weight change only if an additional factor is present while the flag is set. This third factor, signaling reward, punishment, surprise, or novelty, could be implemented by the phasic activity of neuromodulators or specific neuronal inputs signaling special events. While the theoretical framework has been developed over the last decades, experimental evidence in support of eligibility traces on the time scale of seconds has been collected only during the last few years. Here we review, in the context of three-factor rules of synaptic plasticity, four key experiments that support the role of synaptic eligibility traces in combination with a third factor as a biological implementation of neoHebbian three-factor learning rules.

## 1. Introduction

Humans are able to learn novel behaviors such as pressing a button, swinging a tennis racket, or braking at a red traffic light; they are also able to form memories of salient events, learn to distinguish flowers, and to establish a mental map when exploring a novel environment. Memory formation and behavioral learning is linked to changes of synaptic connections (Martin et al., 2000). Long-lasting synaptic changes, necessary for memory, can be induced by Hebbian protocols that combine the activation of presynaptic terminals with a manipulation of the voltage or the firing state of the postsynaptic neuron (Lisman, 2003). Traditional experimental protocols of long-term potentiation (LTP) (Bliss and Lømo, 1973; Bliss and Collingridge, 1993), long-term depression (LTD) (Levy and Stewart, 1983; Artola and Singer, 1993) and spike-timing dependent plasticity (STDP) (Markram et al., 1997; Zhang et al., 1998; Sjöström et al., 2001) neglect that additional factors such as neuromodulators or other gating signals might be necessary to permit synaptic changes (Gu, 2002; Reynolds and Wickens, 2002; Hasselmo, 2006). Early STDP experiments that involved neuromodulators mainly focused on tonic bath application of modulatory factors (Pawlak et al., 2010) with the exception of one study in locusts Cassenaer and Laurent (2012). However, from the perspective of formal learning theories, to be reviewed below, the timing of modulatory factors is just as crucial (Schultz and Dickinson, 2000; Schultz, 2002). From the theoretical perspective, STDP under the control of neuromodulators leads to the framework of three-factor learning rules (Xie and Seung, 2004; Legenstein et al., 2008; Vasilaki et al., 2009) where an eligibility trace represents the Hebbian idea of co-activation of pre- and postsynaptic neurons (Hebb, 1949) while modulation of plasticity by additional gating signals is represented generically by a “third factor” (Crow, 1968; Barto, 1985; Legenstein et al., 2008). Such a third factor could represent variables such as “reward minus expected reward” (Williams, 1992; Schultz, 1998; Sutton and Barto, 1998) or the saliency of an unexpected event (Ljunberg et al., 1992; Redgrave and Gurney, 2006).

In an earlier paper (Frémaux and Gerstner, 2016) we reviewed the theoretical literature of, and experimental support for, three-factor rules available by the end of 2013. During recent years, however, the experimental procedures advanced significantly and provided direct physiological evidence of eligibility traces and three-factor learning rules for the first time, making an updated review of three-factor rules necessary. In the following, we—a group of theoreticians—review five experimental papers indicating support of eligibility traces in striatum (Yagishita et al., 2014), cortex (He et al., 2015), and hippocampus (Brzosko et al., 2015, 2017; Bittner et al., 2017). We will close with a few remarks on the paradoxical nature of theoretical predictions in the field of computational neuroscience.

## 2. Hebbian rules vs. three-factor rules

Learning rules describe the change of the strength of a synaptic contact between a presynaptic neuron *j* and a postsynaptic neuron *i*. The strength of an excitatory synaptic contact can be defined by the amplitude of the postsynaptic potential which is closely related to the spine volume and the number of AMPA receptors (Matsuzaki et al., 2001). Synapses contain complex molecular machineries (Lisman, 2003, 2017; Redondo and Morris, 2011; Huganir and Nicoll, 2013), but for the sake of transparency of the arguments, we will keep the mathematical notation as simple as possible and characterize the synapse by two variables only: the first one is the synaptic strength *w*_*ij*_, measured as spine volume or amplitude of postsynaptic potential, and the second one is a synapse-internal variable *e*_*ij*_ which is not directly visible in standard electrophysiological experiments. In our view, the internal variable *e*_*ij*_ represents a metastable transient state of interacting molecules in the spine head or a multi-molecular substructure in the postsynaptic density which serves as a synaptic flag indicating that the synapse is ready for an increase or decrease of its spine volume (Bosch et al., 2014). The precise biological nature of *e*_*ij*_ is not important to understand the theories and experiments that are reviewed below. We refer to *e*_*ij*_ as the “synaptic flag” or the “eligibility trace” and to *w*_*ij*_ as the “synaptic weight,” or “strength” of the synaptic contact. A change of the synaptic flag indicates a ‘candidate weight change’ (Frémaux et al., 2010) whereas a change of *w*_*ij*_ indicates an actual, measurable, change of the synaptic weight. Before we turn to three-factor rules, let us discuss conventional models of Hebbian learning.

### 2.1. Hebbian learning rules

Hebbian learning rules are the mathematical summary of the outcome of experimental protocols inducing long-term potentiation (LTP) or long-term depression (LTD) of synapses. Suitable experimental protocols include strong extracellular stimulation of presynaptic fibers (Bliss and Lømo, 1973; Levy and Stewart, 1983), manipulation of postsynaptic voltage in the presence of presynaptic spike arrivals (Artola and Singer, 1993), or spike-timing dependent plasticity (STDP) (Markram et al., 1997; Sjöström et al., 2001). In all mathematical formulations of Hebbian learning, the synaptic flag variable *e*_*ij*_ is sensitive to the *combination* of presynaptic spike arrival and a postsynaptic variable, such as the voltage at the location of the synapse. Under a Hebbian learning rule, repeated presynaptic spike arrivals at a synapse of a neuron at rest do not cause a change of the synaptic variable. Similarly, an elevated postsynaptic potential in the absence of a presynaptic spike does not cause a change of the synaptic variable. Thus, Hebbian learning always needs two factors for a synaptic change: a factor caused by a presynaptic signal such as glutamate; and a factor that depends on the state of the postsynaptic neuron.

What are these factors? We can think of the presynaptic factor as the time course of glutamate available in the synaptic cleft or bound to the postsynaptic membrane. Note that the term “presynaptic factor” that we will use in the following *does not imply* that the physical location of the presynaptic factor is inside the presynaptic terminal–the factor could very well be located in the postsynaptic membrane as long as it *only* depends on the amount of available neurotransmitters. The postsynaptic factor might be related to calcium in the synaptic spine (Shouval et al., 2002; Rubin et al., 2005), a calcium-related second messenger molecule (Graupner and Brunel, 2007), or simply the voltage at the site of the synapse (Brader et al., 2007; Clopath et al., 2010).

We remind the reader that we always use the index *j* to refer to the presynaptic neuron and the index *i* to refer to the postsynaptic one. For the sake of simplicity, let us call the presynaptic factor *x*_*j*_ (representing the activity of the presynaptic neuron or the amount of glutamate in the synaptic cleft) and the postsynaptic factor *y*_*i*_ (representing the state of the postsynaptic neuron). In a Hebbian learning rule, changes of the synaptic flag *e*_*ij*_ need both *x*_*j*_ and *y*_*i*_

(1) $$\begin{array}{r} \frac{d}{dt}e_{ij}=\eta f_{j}\left(x_{j}\right)g_{i}\left(y_{i}\right)-e_{ij}/\tau_{e} \end{array}$$

where η is the constant learning rate, τ_*e*_ is a decay time constant, *f*_*j*_(*x*_*j*_) is an (often linear) function of the presynaptic activity *x*_*j*_ and *g*_*i*_(*y*_*i*_) is some arbitrary, potentially nonlinear, function of the postsynaptic variable *y*_*i*_. The index *j* of the function *f*_*j*_ and the index *i* of the function *g*_*i*_ indicate that the rules for changing a synaptic flag can depend on the type of pre- and postsynaptic neurons, on the cortical layer and area, but also on some heterogeneity of parameters between one neuron and the next.

According to Equation 1 the synaptic flag *e*_*ij*_ acts as a *correlation detector* between presynaptic activity *x*_*j*_ and the state of the postsynaptic neuron *y*_*i*_. In some models, there is no decay or the decay is considered negligible on the time scale of one experiment (τ_*e*_ → ∞). The flag variable *e*_*ij*_ could be related to a calcium-based coincidence detection mechanism in the spine such as CaMKII (Lisman, 1989; Shouval et al., 2002) or a metastable state of the molecular machinery in the synapse (Bosch et al., 2014).

**Figure 1:**
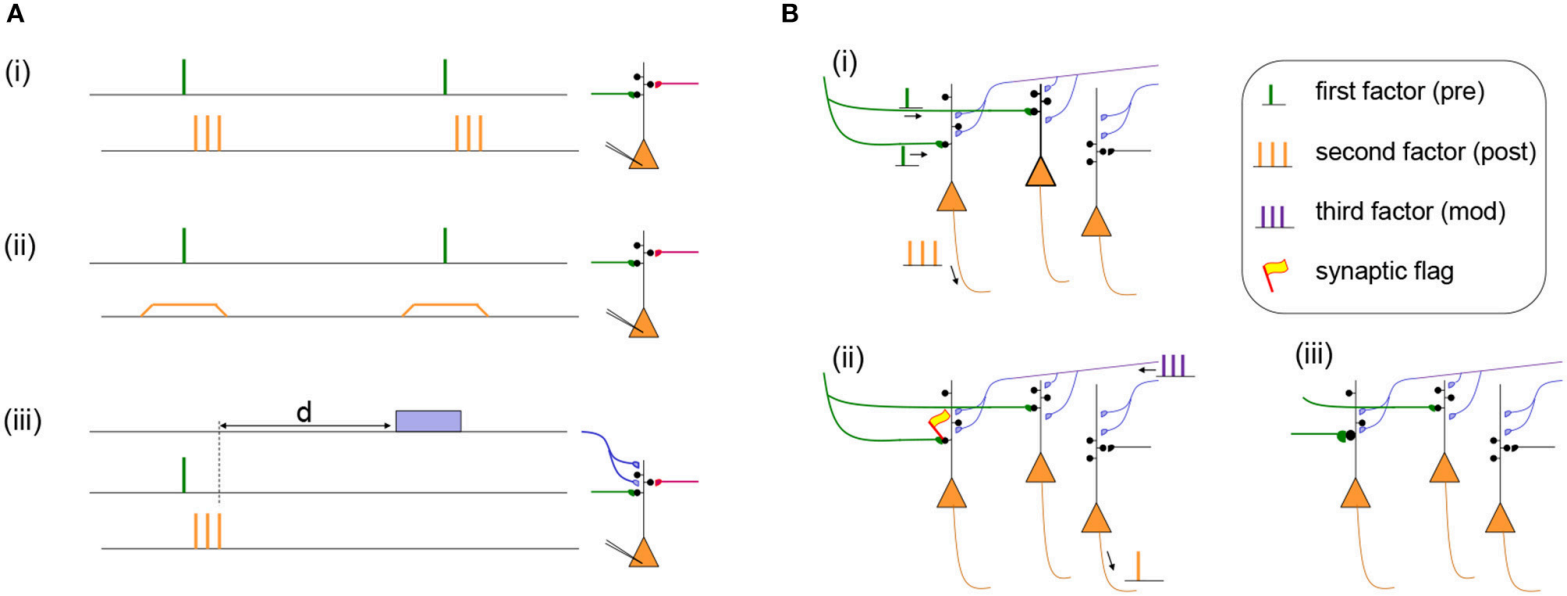
**(A)** Two Hebbian protocols and one three-factor learning protocol. (i) Hebbian STDP protocol with presynaptic spikes (presynaptic factor) followed by a burst of postsynaptic spikes (postsynaptic factor). Synapses in the stimulated pathway (green) will typically show LTP while an unstimulated synapse (red) will not change its weight (Markram et al., 1997). (ii) Hebbian voltage pairing protocol of presynaptic spikes (presynaptic factor) with a depolarization of the postsynaptic neuron (postsynaptic factor). Depending on the amount of depolarization the stimulated pathway (green) will show LTP or LTD while an unstimulated synapse (red) does not change its weight (Artola and Singer, 1993; Ngezahayo et al., 2000). (iii) Results of a Hebbian induction protocol are influenced by a third factor (blue) even if it is given after a delay *d*. The third factor could be a neuromodulator such as dopamine, acetylcholine, noreprinephrine, or serotonin (Pawlak et al., 2010; Yagishita et al., 2014; Brzosko et al., 2015, 2017; He et al., 2015; Bittner et al., 2017). **(B)** Specificity of three-factor learning rules. (i) Presynaptic input spikes (green) arrive at two different neurons, but only one of these also shows postsynaptic activity (orange spikes). (ii) A synaptic flag is set only at the synapse with a Hebbian co-activation of pre- and postsynaptic factors; the synapse become then eligible to interact with the third factor (blue). Spontaneous spikes of other neurons do not interfere. (iii) The interaction of the synaptic flag with the third factor leads to a strengthening of the synapse (green).

Let us discuss two examples. In the Bienenstock-Cooper Munro (BCM) model of developmental cortical plasticity (Bienenstock et al., 1982) the presynaptic factor *x*_*j*_ is the firing rate of the presynaptic neuron and *g*(*y*_*i*_) = (*y*_*i*_−θ)*y*_*i*_ is a quadratic function with *y*_*i*_ the postsynaptic firing rate and θ a threshold rate. Thus, if both pre- and postsynaptic neurons fire together at a high rate *x*_*j*_ = *y*_*i*_>θ then the synaptic flag *e*_*ij*_ increases. In the BCM model, just like in most other conventional models, a change of the synaptic flag (i.e., an internal state of the synapse) leads instantaneously to a change of the weight *e*_*ij*_→*w*_*ij*_ so that an experimental protocol results immediately in a measurable weight change. With the BCM rule and other similar rules (Oja, 1982; Miller and MacKay, 1994), the synaptic weight increases if both presynaptic and postsynaptic neuron are highly active, implementing the slogan “fire together, wire together” (Lowel and Singer, 1992; Shatz, 1992) cf. Figure 1Ai.

As a second example, we consider the Clopath model (Clopath et al., 2010). In this model, there are two correlation detectors implemented as synaptic flags $e_{ij}^{+}$ and $e_{ij}^{-}$ for LTP and LTD, respectively. The synaptic flag $e_{ij}^{+}$ for LTP uses a presynaptic factor $x_{j}^{+}$ (related to the amount of glutamate available in the synaptic cleft) which increases with each presynaptic spike and decays back to zero over the time of a few milliseconds (Clopath et al., 2010). The postsynaptic factor for LTP depends on the postsynaptic voltage *y*_*i*_ via a function $g\left(y_{i}\right)=a_{+}\left[y_{i}-\theta_{+}\right]\bar{y_{i}}$ where *a*_+_ is a positive constant, θ_+_ a voltage threshold, square brackets denote the rectifying piecewise linear function, and $\bar{y_{i}}$ a running average of the voltage with a time constant of tens of milliseconds. An analogous, but simpler, combination of presynaptic spikes and postsynaptic voltage defines the second synaptic flag $e_{ij}^{-}$ for LTD (Clopath et al., 2010). The total change of the synaptic weight is the combination of the two synaptic flags for LTP and LTD: $dw_{ij}/dt=de_{ij}^{+}/dt-de_{ij}^{-}/dt$. Note that, since both synaptic flags $e_{ij}^{+}$ and $e_{ij}^{-}$ depend on the postsynaptic voltage, postsynaptic spikes are not a necessary condition for changes, in agreement with voltage-dependent protocols (Artola and Singer, 1993; Ngezahayo et al., 2000). Thus, in voltage-dependent protocols, and similarly in voltage-dependent models, “wiring together” is possible without “firing together”-indicating that the theoretical framework sketched above goes beyond a narrow view of Hebbian learning; cf. Figure 1Aii.

If we restrict the discussion of the postsynaptic variable to super-threshold spikes, then the Clopath model becomes identical to the triplet STDP model (Pfister et al., 2006) which is in turn closely related to other nonlinear STDP models (Senn et al., 2001; Froemke and Dan, 2002; Izhikevich and Desai, 2003) as well as to the BCM model discussed above (Pfister et al., 2006; Gjorjieva et al., 2011). Classic pair-based STDP models (Gerstner et al., 1996; Kempter et al., 1999; Song et al., 2000; van Rossum et al., 2000; Rubin et al., 2001) are further examples of the general theoretical framework of Equation (1) and so are some models of structural plasticity (Helias et al., 2008; Deger et al., 2012, 2018; Fauth et al., 2015). Hebbian models of synaptic consolidation have several hidden flag variables (Fusi et al., 2005; Barrett et al., 2009; Benna and Fusi, 2016) but can also be situated as examples within the general framework of Hebbian rules. Note that in most of the examples so far the measured synaptic weight is a linear function of the synaptic flag variable(s). However, this does not need to be the case. For example, in some voltage-based (Brader et al., 2007) or calcium-based models (Shouval et al., 2002; Rubin et al., 2005), the synaptic flag is transformed into a weight change only if *e*_*ij*_ is above or below some threshold, or only after some further filtering.

To summarize, in the theoretical literature the class of Hebbian models is a rather general framework encompassing all those models that are driven by a combination of presynaptic activity and the state of the postsynaptic neuron. In this view, Hebbian models depend on two factors related to the activity of the presynaptic and the state of the postsynaptic neuron. The correlations between the two factors can be extracted on different time scales using one or, if necessary, several flag variables. The flag variables trigger a change of the measured synaptic weight. In the following we build on Hebbian learning, but extend the theoretical framework to include a third factor.

### 2.2. Three-factor learning rules

We are interested in a framework where a Hebbian co-activation of two neurons leaves one or several flags (eligibility trace) at the synapse connecting these neurons. The flag is not directly visible and does not automatically trigger a change of the synaptic weight. An actual weight change is implemented only if a third signal, e.g., a phasic increase of neuromodulator activity or an additional input (signaling the occurrence of a special event) is present at the same time or in the near future. Theoreticians refer to such a plasticity model as a three-factor learning rule (Xie and Seung, 2004; Legenstein et al., 2008; Vasilaki et al., 2009; Frémaux et al., 2013; Frémaux and Gerstner, 2016). Three-factor rules have also been called “neoHebbian” (Lisman et al., 2011; Lisman, 2017) or “heterosynaptic (modulatory-input dependent)” (Bailey et al., 2000) and can be traced back to the 1960s (Crow, 1968), if not earlier. To our knowledge the wording “three factors” was first used by (Barto, 1985). The terms eligibility and eligibility traces have been used in (Klopf, 1972; Sutton and Barto, 1981, 1998; Barto et al., 1983; Barto, 1985; Williams, 1992; Schultz, 1998) but in some of the early studies it remained unclear whether eligibility traces can be set by presynaptic activity alone (Klopf, 1972; Sutton and Barto, 1981) or only by Hebbian co-activation of pre- and postsynaptic neurons (Barto et al., 1983; Barto, 1985; Williams, 1992; Schultz, 1998; Sutton and Barto, 1998).

The basic idea of a modern eligibility trace is that a synaptic flag variable *e*_*ij*_ is set according to Equation (1) by coincidences between presynaptic activity *x*_*j*_ and a postsynaptic factor *y*_*i*_. The update of the synaptic weight *w*_*ij*_, as measured via the spine volume or the amplitude of the excitatory postsynaptic potential (EPSP), is given by

(2) $$\begin{array}{r} \frac{d}{dt}w_{ij}=e_{ij}M^{3rd}\left(t\right) \end{array}$$

where *M*^3*rd*^(*t*) refers to the global third factor (Izhikevich, 2007; Legenstein et al., 2008; Frémaux et al., 2013). Therefore, according to Equation 2, a non-zero third factor is needed to transform the eligibility trace into a weight change; cf. Figure 1Aiii. Note that the weight change is proportional to *M*^3*rd*^(*t*). Thus, the third factor influences the speed of learning. In the absence of the third factor (*M*^3*rd*^(*t*) = 0), the synaptic weight is not changed. We emphasize that a positive value of the synaptic flag in combination with a negative value *M*^3*rd*^ < 0 leads to a decrease of the weight. Therefore, the third factor also influences the *direction* of change.

In the discussion so far, *M*^3*rd*^(*t*) in Equation (2) can take positive and negative values. Such a behavior is typical for a phasic signal which we may mathematically define as the deviation from a running average. We may, for example, think of the third factor as a phasic neuromodulatory signal. However, the third factor could also be biologically implemented by *positive* excursions of the activity using two different neuromodulators with very low baseline activity. The activity of the first modulator could indicate positive values of the third factor and that of the second modulator negative ones - similar to ON and OFF cells in the retina. Similarly, the framework of neoHebbian three-factor rules is general enough to enable biological implementations with separate eligibility traces for LTP and LTD as discussed above in the context of the Clopath model (Clopath et al., 2010).

What could be the source of such a third factor, be it a single neuromodulator or several different ones? The third factor could be triggered by attentional processes, surprising events, or reward. Phasic signals of neuromodulators such as dopamine, serotonin, acetylcholine, or noradrenaline are obvious candidates for a third factor, but potentially not the only ones. Note that axonal branches of most dopaminergic, serotonergic, cholinergic, or adrenergic neurons project broadly onto large regions of cortex so that a phasic neuromodulator signal arrives at many neurons and synapses in parallel (Schultz, 1998). Since neuromodulatory information is shared by many neurons, the variable *M*^3*rd*^(*t*) of the third factor has no neuron-specific index (neither *i* nor *j*) in our mathematical formulation. Because of its nonspecific nature, the theory literature sometimes refers to the third factor as a “global” broadcasting signal, even though in practice not every brain region and every synapse is reached by each neuromodulator. The learning rule with the global modulator, as formulated in Equation 2 will be called Type 1 for the remainder of this paper.

To account for some neuron-specific aspects, three-factor learning rules of Type 2,

(3) $$\begin{array}{r} \frac{d}{dt}w_{ij}=e_{ij}h_{i}\left(M^{3rd}\left(t\right)\right), \end{array}$$

contain a neuron-specific function *h*_*i*_ that determines how the global third factor *M*^3*rd*^ influences synaptic plasticity of the postsynaptic neuron *i*. Including an index *i* in the function $h_{i}\left(M^{3rd}\right)$ keeps the theory flexible enough to set (for example) $h_{i}\left(M^{3rd}\right)\equiv 0$ for the subset of neurons *i* that are not reached by a given neuromodulator. In this case, the classification of a given learning rule as Type 1 and Type 2 is somewhat arbitrary as it depends on whether a population of neurons with a three-factor learning rule is embedded in a larger network with static synapses or not. But there are also existing models, where the implementation of a certain function requires the possibility that one subpopulation has plasticity rules under a third factor whereas another one does not (Brea et al., 2013; Rezende and Gerstner, 2014).

The framework of Equation 3 also includes networks where the distribution of neuromodulatory information to different neurons is done with fixed, but random feedback weights *b*_*i*_ (Lillicrap et al., 2016; Guerguiev et al., 2017); we simply need to set $h_{i}\left(M^{3rd}\right)=h\left(b_{i}M^{3rd}\right)$. It does not, however, include the general case of supervised learning with backpropagation of errors.

One of the important differences between supervised and reinforcement learning is that in most modern supervised learning tasks, such as auto-encoders, the target is, just like the input, a high-dimensional vector. For supervised learning of high-dimensional targets, we need to generalize Equation (3) further, to three-factor learning rules of Type 3,

(4) $$\begin{array}{r} \frac{d}{dt}w_{ij}=e_{ij}M_{i}^{3rd}\left(t\right), \end{array}$$

where $M_{i}^{3rd}$ is now a neuron-specific (hence non-global) error feedback signal. For the case of standard backpropagation in layered networks, $M_{i}^{3rd}$ is calculated by a weighted sum over the errors in the next layer closer to the output; a calculation which needs a well-tuned feedback circuit from the output back to previous layers (Roelfsema and van Ooyen, 2005; Lillicrap et al., 2016; Roelfsema and Holtmaat, 2018). Interestingly, learning similar, but not identical, to backpropagation is still possible with feedback circuits, where the direct feedback from the output is replaced with fixed random weights (Lillicrap et al., 2016; Guerguiev et al., 2017), or in networks with a single hidden layer and winner-take-all activity (one-hot coding) in the output layer (Roelfsema and van Ooyen, 2005; Rombouts et al., 2015). In the latter case, the neuron-specific third factor $M_{i}^{3rd}$ further factorizes into a global modulator *M*^3*rd*^ and an attention signal *A*_*i*_, which leads to a four-factor learning rule (Roelfsema and Holtmaat, 2018).

### 2.3. Examples and theoretical predictions

There are several known examples in the theoretical literature of neoHebbian three-factor rules. We briefly present four of these and formulate expectations derived from the theoretical framework which we would like to compare to experimental results in the next section.

#### 2.3.1. Reward-based learning

As a first example of a Type 1 three-factor learning rule, we consider the relation of neoHebbian three-factor rules to reward-based learning. Temporal Difference (TD) algorithms such as SARSA(λ) or TD(λ) from the theory of reinforcement learning (Sutton and Barto, 1998) as well as learning rules derived from policy gradient theories (Williams, 1992) can be interpreted in neuronal networks in terms of neoHebbian three-factor learning rules. The resulting plasticity rules are applied to synapses connecting “state-neurons” (e.g., place cells coding for the current location of an animal) to “action neurons” e.g., cells initiating an action program such as “turn left”) (Brown and Sharp, 1995; Suri and Schultz, 1999; Arleo and Gerstner, 2000; Foster et al., 2000; Xie and Seung, 2004; Loewenstein and Seung, 2006; Florian, 2007; Izhikevich, 2007; Legenstein et al., 2008; Vasilaki et al., 2009; Frémaux et al., 2013); for a review, see (Frémaux and Gerstner, 2016). The eligibility trace is increased during the joint activation of “state-neurons” and “action-neurons” and decays exponentially thereafter consistent with the framework of Equation (1). The third factor is defined as reward minus expected reward where the exact definition of expected reward depends on the implementation details. A long line of research by Wolfram Schultz and colleagues (Schultz et al., 1997; Schultz, 1998, 2002; Schultz and Dickinson, 2000) indicates that phasic increases of the neuromodulator dopamine have the necessary properties required for a third factor in the theoretical framework of reinforcement learning.

However, despite the rich literature on dopamine and reward-based learning accumulated during the last 25 years, there is scant data on the decay time constant τ_*e*_ of the eligibility trace *e*_*ij*_ in Equation (1) before 2015 (except the locusts study Cassenaer and Laurent, 2012). From the mathematical framework of neoHebbian three-factor rules it is clear that, in the context of action learning, the time constant of the eligibility trace (i.e., the duration of the synaptic flag) should roughly match the time span from the initiation of an action to the delivery of reward. As an illustration, let us imagine a baby that attempts to grasp her bottle of milk. The typical duration of one grasping movement is in the range of a second, but potentially only the third grasping attempt might be successful. Let us suppose that each grasping movement corresponds to the co-activation of some neurons in the brain. If the duration of the synaptic flag is much less than a second, the co-activation of pre- and postsynaptic neurons that sets the synaptic flag (eligibility trace) cannot be linked to the reward 1 s later and synapses do not change. If the duration of the synaptic flag is much longer than a second, then the two “wrong” grasping attempts are reinforced nearly as strongly as the third, successful one which mixes learning of “wrong” co-activations with the correct ones. Hence, *the existing theory of three-factor learning rules predicts that the synaptic flag (eligibility trace for action learning) should be in the range of a typical elementary action, about 200 ms to 2 s*; see, for example, p. 15 of Schultz (1998)^1^, p.3 of Izhikevich (2007) ^2^, p.3 of Legenstein et al. (2008)^3^, p. 13327 of Frémaux et al. (2010)^4^, or p. 13 of Frémaux et al. (2013)^5^. An eligibility trace of <100 ms or more than 10 s would be less useful for learning a typical elementary action or delayed reward task than an eligibility trace in the range of 200 ms to 2 s. The expected time scale of the synaptic eligibility trace should roughly match the maximal delay of reinforcers in conditioning experiments (Thorndike, 1911; Pavlov, 1927; Black et al., 1985), linking synaptic processes to behavior. For human behavior, delaying a reinforcer by 10 s during ongoing actions decreases learning compared to immediate reinforcement (Okouchi, 2009).

#### 2.3.2. Surprise-based learning

As a second example of a Type 1 three-factor learning rule, we consider situations that go beyond standard reward-based learning. Even in the absence of reward, a surprising event might trigger a combination of neuromodulators such as noradrenaline, acetylcholine and dopamine that may act as third factor for synaptic plasticity. Imagine a small baby lying in the cradle with an attractive colorful object swinging above him. He spontaneously makes several arm movements until finally he succeeds, by chance, to grasp the object. There is no food reward for this action. However, the fact that he can now turn the object, look at it from different sides, or put it in his mouth is satisfying because it leads to many novel (and exciting!) stimuli. The basic idea is that, in such situations, novelty or surprise acts as a reinforcer even in complete absence of food rewards (Schmidhuber, 1991; Singh et al., 2004; Oudeyer et al., 2007). Theoreticians have studied these ideas in the context of curiosity (Schmidhuber, 2010), information gain during active exploration (Storck et al., 1995; Schmidhuber, 2006; Sun et al., 2011; Little and Sommer, 2013; Friston et al., 2016), and via formal definitions of surprise (Shannon, 1948; Storck et al., 1995; Itti and Baldi, 2009; Friston, 2010; Schmidhuber, 2010; Faraji et al., 2018). Note that surprise is not always linked to active exploration but can also occur in a passive situation, e.g., listening to tone beeps or viewing simple stimuli (Squires et al., 1976; Kolossa et al., 2013, 2015; Meyniel et al., 2016). Measurable physiological responses to surprise include pupil dilation (Hess and Polt, 1960) and the P300 component of the electroencephalogram (Squires et al., 1976).

If surprise can play a role similar to reward, then surprise-transmitting broadcast signals should speed-up plasticity. Indeed, theories of surprise as well as hierarchical Bayesian models predict a faster change of model parameters for surprising stimuli than for known ones (Yu and Dayan, 2005; Nassar et al., 2010; Mathys et al., 2011, 2014; Faraji et al., 2018) similar to, but more general than, the well-known Kalman filters (Kalman, 1960). Since the translation of these abstract models into networks of spiking neurons is still missing, precise predictions for surprise modulation of plasticity in the form of three-factor rules are not yet available. However, if we consider noradrenaline, acetylcholine, and/or dopamine as candidate neuromodulators signaling novelty and surprise, we expect that these neuromodulators should have a strong effect on plasticity so as to boost learning of surprising stimuli. The influence of tonic applications of various neuromodulators on synaptic plasticity has been shown in numerous studies (Gu, 2002; Reynolds and Wickens, 2002; Hasselmo, 2006; Pawlak et al., 2010). However, in the context of the above examples, we are interested in phasic neuromodulatory signals. Phasic signals conveying moments of surprise are most useful for learning if they are either synchronous with the stimulus to be learned (e.g., passive listening or viewing) or arise with a delay corresponding to one exploratory movement (e.g., grasping). Hence, we predict from these considerations a decay constant τ_*e*_ of the synaptic flag in the range of 1 s, but with a pronounced effect for synchronous or near-synchronous events.

#### 2.3.3. Synaptic tagging-and-capture

As our third example of a Type 1 three-factor learning rule, we would like to comment on synaptic consolidation. The synaptic tagging-and-capture hypothesis (Frey and Morris, 1997; Reymann and Frey, 2007; Redondo and Morris, 2011) perfectly fits in the framework of three-factor learning rules: The joint pre- and postsynaptic activity sets the synaptic flag (called “tag” in the context of consolidation) which decays back to zero over the time of 1 h. To stabilize synaptic weights beyond 1 h an additional factor is needed to trigger protein synthesis required for long-term maintenance of synaptic weights (Reymann and Frey, 2007; Redondo and Morris, 2011). Neuromodulators such as dopamine have been identified as the necessary third factor for consolidation (Bailey et al., 2000; Reymann and Frey, 2007; Redondo and Morris, 2011; Lisman, 2017). Indeed, modern computational models of synaptic consolidation take into account the effect of neuromodulators (Clopath et al., 2008; Ziegler et al., 2015) in a framework reminiscent of the three-factor rule defined by Equations (1, 2) above. However, there are two noteworthy differences. First, in contrast to reward-based learning, the decay time τ_*e*_ of the synaptic tag *e*_*ij*_ is in the range of 1 h rather than 1 s, consistent with slice experiments (Frey and Morris, 1997) as well as with behavioral experiments (Moncada and Viola, 2007). Second, in slices, the measured synaptic weights *w*_*ij*_ are increased a few minutes after the end of the induction protocol and decay back with the time course of the synaptic tag whereas in the simplest implementation of the three-factor rule framework as formulated in Equations (1, 2) the visible weight is only updated at the moment when the third factor is present. However, slightly more involved models where the visible weight depends on both the synaptic tag variable and the long-term stable weight (Clopath et al., 2008; Ziegler et al., 2015) correctly account for the time course of the measured synaptic weights in consolidation experiments (Frey and Morris, 1997; Reymann and Frey, 2007; Redondo and Morris, 2011).

#### 2.3.4. Supervised learning with segregated dendrites

A recent study proposes a mechanism for implementing 3-factor rules of Type 3 in the context of supervised learning (Guerguiev et al., 2017). Instead of neuromodulators, they propose that top-down feedback signals from the network output to the apical dendrites of pyramidal neurons serve as the 3rd factor in the 3-factor rule. If the output units have a stationary value *y*_*k*_ when driven by the feedforward network input and a stationary value ŷ_*k*_ when shunted to the target value, then the changes of a weight *w*_*ij*_ from neuron *j* onto the soma (or basal dendrites) of neuron *i* is governed by Equation (4) with a third factor $M_{i}=\underset{k}{\sum }b_{ik}\left({ŷ}_{k}-y_{k}\right)$ where *b*_*ik*_ are random feedback weights from the output neuron *k* to the apical dendrite of neuron *i* (Guerguiev et al., 2017). The authors assume relatively weak electrical coupling between the apical dendrite and the soma and suggest that bursts in the apical dendrite could transmit the value of the third factor to synapses onto the soma or basal dendrites (Guerguiev et al., 2017).

Similarly, the Urbanczik-Senn rule for supervised learning can be interpreted as a three-factor rule of Type 3 (Urbanczik and Senn, 2014). Target input to a neuron in the output layer is given at the soma and leads to a spike-train *S*_*i*_(*t*) while feedforward input from other neurons in the network arrives in a dendritic compartment where it generates a voltage *y*_*i*_. The third factor $M_{i}^{3rd}\left(t\right)=S_{i}\left(t\right)-\phi \left(y_{i}\left(t\right)\right)$ compares the actual spike train (including the somatic drive by the target) with the firing rate expected from dendritic input alone (Urbanczik and Senn, 2014). The authors assume relatively strong electrical coupling between the dendrite and the soma. Interestingly, the same learning rule can also be used in the absence of target information; in this case we prefer to interpret it as a Hebbian two-factor rule, as discussed in the next paragraph.

#### 2.3.5. Summary

The Clopath rule discussed in the paragraph on Hebbian learning rules contains terms that combine a presynaptic factor with two postsynaptic factors, one for instantaneous superthreshold voltage and the other one for low-pass filtered voltage (Clopath et al., 2010). However, despite the fact that it is possible to write the Clopath rule as a multiplication of three factors, we do not classify it as a three-factor rule but rather as a two-factor rule with a nonlinear postsynaptic factor. The main difference to a true three-factor rule is that the third factor *M*^3*rd*^ should be related to a feedback signal conveying information on the performance of the network as a whole. As we have seen, this third factor can be a global scalar signal related to reward or surprise or a neuron-specific signal related to the error in the network output. With this nomenclature, the Urbanczik-Senn rule is, just like the Clopath rule (Clopath et al., 2010), a Hebbian two-factor rule if used in unsupervised learning (Urbanczik and Senn, 2014), but the same rule must be seen as a three-factor rule with a neuron-specific (non-global) third factor in the supervised setting.

In summary, the neoHebbian three-factor rule framework has a wide range of applicability. The framework is experimentally well-established in the context of synaptic consolidation where the duration of the flag (“synaptic tag”) extracted from slice experiments (Frey and Morris, 1997) is in the range of 1 h, consistent with fear conditioning experiments (Moncada and Viola, 2007). This time scale is significantly longer than what is needed for behavioral learning of elementary actions or for memorizing surprising events. In the context of reward-based learning, theoreticians therefore hypothesized that a process analogous to setting a tag (“eligibility trace”) must also exist on the time scale of 1 s. The next section discusses some recent experimental evidence supporting this theoretical prediction.

## 3. Experimental evidence for eligibility traces

Recent experimental evidence for eligibility traces in striatum (Yagishita et al., 2014), cortex (He et al., 2015), and hippocampus (Brzosko et al., 2015, 2017; Bittner et al., 2017) is reviewed in the following three subsections.

### 3.1. Eligibility traces in dendritic spines of medium spiny striatal neurons in nucleus accumbens

**Figure 2:**
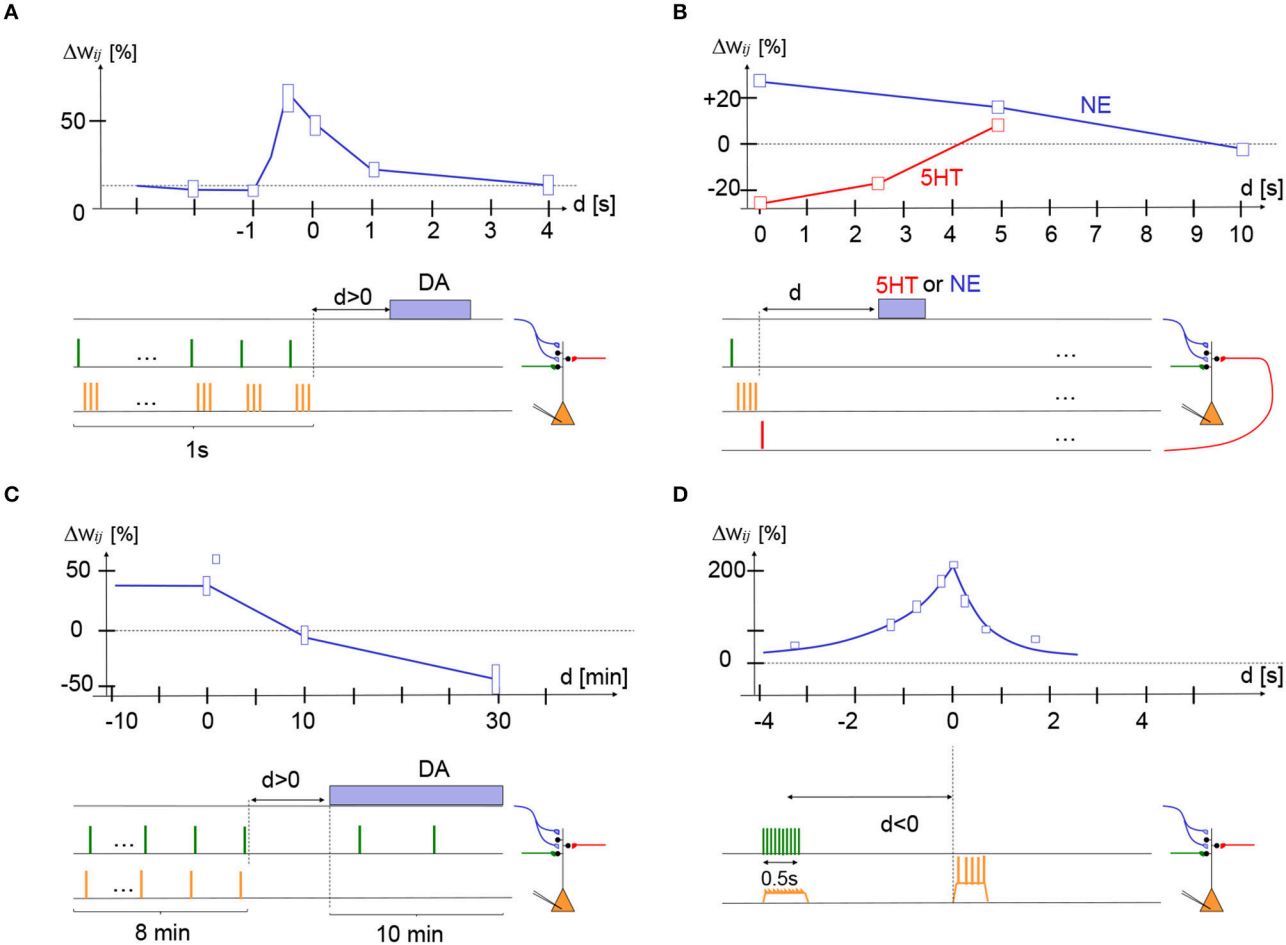
Experimental support for synaptic eligibility traces. Fractional weight change (vertical axis) as a function of delay *d* of third factor (horizontal axis) for various protocols (schematically indicated at the bottom of each panel). **(A)** In striatum medium spiny cells, stimulation of presynaptic glutamatergic fibers (green) followed by three postsynaptic action potentials (STDP with pre-post-post-post at +10 ms) repeated 10 times at 10 Hz yields LTP if dopamine fibers are stimulated during the presentation (*d* < 0) or shortly afterward (*d* = 0 s or *d* = 1 s) but not if dopamine is given with a delay *d* = 4 s; redrawn after Figure 1 of Yagishita et al. (2014), with delay *d* defined as time since end of STDP protocol. **(B)** In cortical layer 2/3 pyramidal cells, stimulation of two independent presynaptic pathways (green and red) from layer 4 to layer 2/3 by a single pulse combined with a burst of four postsynaptic spikes (orange). If the pre-before-post stimulation was combined with a pulse of norepinephrine (NE) receptor agonist isoproterenol with a delay of 0 or 5 s, the protocol gave LTP (blue trace). If the post-before-pre stimulation was combined with a pulse of serotonin (5-HT) of a delay of 0 or 2.5 s, the protocol gave LTD (red trace); redrawn after Figure 6 of He et al. (2015). **(C)** In hippocampus CA1, a post-before-pre (Δ*t* = -20 ms) induction protocol yields LTP if dopamine is present during induction or given with a delay *d* of 0 or 1 min, but yields LTD if dopamine is absent or given with a delay of 30 min; redrawn after Figures 1F, 2B, and 3C (square data point at delay of 1 min) of Brzosko et al. (2015). **(D)** In hippocampus CA1, 10 extracellular stimuli of presynaptic fibers at 20 Hz cause depolarization of the postsynaptic potential. The timing of a complex spike (calcium plateau potential) triggered by current injection (during 300 ms) after a delay *d*, is crucial for the amount of LTP. If we interpret presynaptic spike arrival as the first, and postsynaptic depolarization as the second factor, the complex spike could be associated with a third factor; redrawn after Figure 3 of Bittner et al. (2017). Height of boxes gives a very rough estimate of standard deviation - see original papers and figures for details.

In their elegant imaging experiment of dendritic spines of nucleus accumbens neurons, Yagishita et al. (2014) mimicked presynaptic spike arrival by glutamate uncaging (presynaptic factor), paired it with three postsynaptic spikes immediately afterward (postsynaptic factor), repeated this STDP-like pre-before-post sequence ten times, and combined it with optogenetic stimulation of dopamine fibers (3rd factor) at various delays (Yagishita et al., 2014). The ten repetitions of the pre-before-post sequence at 10 Hz took about 1 s while stimulation of dopaminergic fibers (10 dopamine pulses at 30 Hz) projecting from the ventral tegmental area (VTA) to nucleus accumbens took about 0.3 s. In their paper, dopamine was counted as delayed by 1 s if the dopamine stimulation started immediately after the end of the 1 s-long induction period (delay = difference in switch-on time of STDP and dopamine), but for consistency with other data we define the delay *d* here as the time passed since the end of the STDP protocol. After 15 complete trials the spine volume, an indicator of synaptic strength (Matsuzaki et al., 2001), was measured and compared with the spine volume before the induction protocol. The authors found that dopamine promoted spine enlargement only if phasic dopamine was given in a narrow time window during or immediately after the 1 s-long STDP protocol; cf. Figure 2A.

The maximum enlargement of spines occurred if the dopamine signal started during the STDP protocol (*d* = −0.4 s), but even at a delay of *d* = 1 s LTP was still visible. Giving dopamine too early (*d* = −2 s) or too late (*d* = +4 s) had no effect. Spine enlargement corresponded to an increase in the amplitude of excitatory postsynaptic currents indicating that the synaptic weight was indeed strengthened after the protocol (Yagishita et al., 2014). Thus, we can summarize that we have in the *striatum a three-factor learning rule for the induction of LTP where the decay of the eligibility trace occurs on a time scale of 1 s*; cf. Figure 2A.

To arrive at these results, Yagishita et al. (2014) concentrated on medium spiny neurons in the nucleus accumbens core, a part of the ventral striatum of the basal ganglia. Functionally, striatum is a particularly interesting candidate for reinforcement learning (Brown and Sharp, 1995; Schultz, 1998; Arleo and Gerstner, 2000; Doya, 2000a; Daw et al., 2005) for several reasons. First, striatum receives highly processed sensory information from neocortex and hippocampus through glutamatergic synapses (Mink, 1996; Middleton and Strick, 2000; Haber et al., 2006). Second, striatum also receives dopamine input associated with reward processing (Schultz, 1998). Third, striatum is, together with frontal cortex, involved in the selection of motor action programs (Mink, 1996; Seo et al., 2012).

On the molecular level, the striatal three-factor plasticity depended on NMDA, CaMKII, protein synthesis, and dopamine D1 receptors (Yagishita et al., 2014; Shindou et al., 2018). CaMKII increases were found to be localized in the spine and to have roughly the same time course as the critical window for phasic dopamine suggesting that CaMKII could be involved in the “synaptic flag” triggered by the STDP-like induction protocol, while protein kinase A (PKA) was found to have a nonspecific cell-wide distribution suggesting an interpretation of PKA as a molecule linked to the dopamine-triggered third factor (Yagishita et al., 2014).

### 3.2. Two distinct eligibility traces for LTP and LTD in cortical synapses

In a recent experiment of He et al. (2015), layer 2/3 pyramidal cells in slices from prefrontal or visual cortex were stimulated by an STDP protocol, either pre-before-post for LTP induction or post-before-pre for LTD induction. A neuromodulator was applied with a delay after a single STDP sequence before the whole protocol was repeated; cf. Figure 2B. Neuromodulators, either norepinephrine (NE), serotonin (5-HT), dopamine (DA), or acetylcholine (ACh) were ejected from a pipette for 10 s or from endogenous fibers (using optogenetics) for 1 s (He et al., 2015). It was found that NE was necessary for LTP whereas 5-HT was necessary for LTD. DA or ACh agonists had no effect in visual cortex but DA had a positive effect on LTP induction in frontal cortex (He et al., 2015).

For the STDP protocol, He et al. (2015) used extracellular stimulation of two presynaptic pathways from layer 4 to layer 2/3 (presynaptic factor) combined with a burst of 4 postsynaptic action potentials (postsynaptic factor), either pre-before-post or post-before-pre. In a first variant of the experiment, the STDP stimulation was repeated 200 times at 10 Hz corresponding to a total stimulation time of 20 s before the NE or 5-HT was given. In a second variant, instead of an STDP protocol, they paired presynaptic stimulation (first factor) with postsynaptic depolarization (second factor) to –10 mV to induce LTP, or to –40 mV to induce LTD. With both protocols it was found that LTP can be induced if the neuromodulator NE (third factor) arrived with a delay of 5 s or less after the LTP protocol, but not 10 s. LTD could be induced if 5-HT (third factor) arrived with a delay of 2.5 s or less after the LTD protocol, but not 5 s (He et al., 2015).

A third variant of the experiment involved optogenetic stimulation of the noradrenaline, dopamine, or serotonin pathway by repeated light pulses during 1 s applied immediately, or a few seconds, after a minimal STDP protocol consisting of a single presynaptic and four postsynaptic pulses (either pre-before-post or post-before-pre), a protocol that is physiologically more plausible. The minimal sequence of STDP pairing and neuromodulation was repeated 40 times at intervals of 20 s. Results with optogenetic stimulation were consistent with those mentioned above and showed in addition that application of NE or 5-HT immediately before the STDP stimulus did not induce LTP or LTD. *Overall these results indicate that in visual and frontal cortex, pre-before-post pairing leaves an eligibility trace that decays over 5–10 s and that can be converted into LTP by the neuromodulator noradrenaline. Similarly, post-before-pre pairing leaves a shorter eligibility trace that decays over 3 s and can be converted into LTD by the neuromodulator serotonin*; cf. Figure 2B.

Functionally, a theoretical model in the same paper (He et al., 2015) showed that the measured three-factor learning rules with two separate eligibility traces stabilized and prolonged network activity so as to allow “event prediction.” The authors hypothesized that these three-factor rules were related to reward-based learning in cortex such as perceptual learning in monkeys (Schoups et al., 2001) or mice (Poort et al., 2015) or reward prediction (Shuler and Bear, 2006). The relation to surprise was not discussed but might be a direction for further explorations.

Molecularly, the transformation of the Hebbian pre-before-post eligibility trace into LTP involves beta adrenergic receptors and intracellular cyclic adenosine monophosphate (cAMP) whereas the transformation of the post-pre eligibility trace into LTD involves the 5-HT_2*c*_ receptor (He et al., 2015). Both receptors are anchored at the postsynaptic density consistent with a role in the transformation of an eligibility trace into actual weight changes (He et al., 2015).

### 3.3. Eligibility traces in hippocampus

Two experimental groups studied eligibility traces in CA1 hippocampal neurons using complementary approaches. In the studies of Brzosko et al. (2015, 2017), CA1 neurons in hippocampal slices were stimulated during about 8 min in an STDP protocol involving 100 repetitions (at 0.2 Hz) of pairs of one extracellularly delivered presynaptic stimulation pulse (presynaptic factor) and one postsynaptic action potential (postsynaptic factor) (Brzosko et al., 2015). Repeated pre-before-post with a relative timing +10 ms gave LTP (in the presence of natural endogenous dopamine) whereas post-before-pre (–20 ms) gave LTD. However, with additional dopamine (third factor) in the bathing solution, post-before-pre at –20 ms gave LTP (Zhang et al., 2009). Similarly, an STDP protocol with post-before-pre at –10 ms resulted in LTP when endogenous dopamine was present, but in LTD when dopamine was blocked (Brzosko et al., 2015). Thus dopamine broadens the STDP window for LTP into the post-before-pre regime (Zhang et al., 2009; Pawlak et al., 2010). Moreover, in the presence of ACh during the STDP stimulation protocol, pre-before-post at +10ms also gave LTD (Brzosko et al., 2017). Thus ACh broadens the LTD window.

The crucial experiment of Brzosko et al. (2015) involved a delay in the dopamine (Brzosko et al., 2015). Brzosko et al. started to perfuse dopamine either immediately after the end of the post-before-pre (-20ms) induction protocol or with a delay. Since the dopamine was given for about 10 min, it cannot be considered as a phasic signal – but at least the *start* of the dopamine perfusion was delayed. Brzosko et al. found that the stimulus that would normally have given LTD turned into LTP if the delay of dopamine was in the range of 1 min or less, but not if dopamine started 10 min after the end of the STDP protocol (Brzosko et al., 2015). Note that for the conversion of LTD into LTP, it was important that the synapses were weakly stimulated at low rate while dopamine was present. Similarly, a prolonged pre-before-post protocol at +10 ms in the presence of ACh gave rise to LTD, but with dopamine given with a delay of <1 min the same protocol gave LTP (Brzosko et al., 2017). To summarize, *in the hippocampus a prolonged post-before-pre protocol (or a pre-before-post protocol in the presence of ACh) yields visible LTD, but also sets an invisible synaptic flag for LTP. If dopamine is applied with a delay of*<*1 min, the synaptic flag is converted into a positive weight change under continued weak presynaptic stimulation*; cf. Figure 2C.

Molecularly, the conversion of LTD into LTP after repeated stimulation of post-before-pre pulse pairings depended on NMDA receptors and on the cAMP - PKA signaling cascade (Brzosko et al., 2015). The source of dopamine could be in the Locus Coeruleus which would make a link to arousal and novelty (Takeuchi et al., 2016) or from other dopamine nuclei linked to reward (Schultz, 1998). Since the time scale of the synaptic flag reported in Brzosko et al. (2015, 2017) was in the range of minutes, the process studied by Brzosko et al. could be related to synaptic consolidation (Frey and Morris, 1997; Reymann and Frey, 2007; Redondo and Morris, 2011; Lisman, 2017) rather than eligibility traces in reinforcement learning where shorter time constants are needed (Izhikevich, 2007; Legenstein et al., 2008; Frémaux et al., 2010, 2013). The computational study in Brzosko et al. (2015) used an eligibility trace with a time constant of 2 s and showed that dopamine as a reward signal induced learning of reward location while ACh during exploration enabled a fast relearning after a shift of the reward location (Brzosko et al., 2017).

The second study combined *in vivo* with *in vitro* data (Bittner et al., 2017). From *in vivo* studies it has been known that CA1 neurons in mouse hippocampus can develop a novel, reliable, and rather broadly tuned, place field in a single trial under the influence of a “calcium plateau potential” (Bittner et al., 2015), visible as a complex spike at the soma. Moreover, an artificially induced complex spike was sufficient to induce such a novel place field *in vivo* (Bittner et al., 2015, 2017).

In additional slice experiments, several input fibers from CA3 to CA1 neurons were stimulated by 10 pulses from an extracellular electrode during 1 s. The resulting nearly synchronous inputs at, probably, multiple synapses caused a total EPSP that was about 10 mV above baseline at the soma, and potentially somewhat larger in the dendrite, but did not cause somatic spiking of the CA1 neuron. The stimulated synapses showed LTP if the presynaptic stimulation was paired with a calcium plateau potential (complex spike) in the postsynaptic neuron. LTP occurred, even if the presynaptic stimulation stopped 1 or 2 s before the start of the plateau potential or if the plateau potential started before the presynaptic stimulation (Bittner et al., 2017). The protocol has a remarkable efficiency since potentiation was around 200% after only 5 pairings. Thus, *the joint activation of many synapses sets a flag at the activated synapses which is translated into LTP if a calcium plateau potential (complex spike) occurs a few seconds before or after the synaptic activation*; cf. Figure 2D. Molecularly, the plasticity processes implied NMDA receptors and calcium channels (Bittner et al., 2017).

Functionally, synaptic plasticity in hippocampus is particularly important because of the role of hippocampus in spatial memory (O'Keefe and Nadel, 1978). CA1 neurons get input from CA3 neurons which have a narrow place field. The emergence of a broad place field in CA1 has therefore been interpreted as linking several CA3 neurons (that cover for example the 50 cm of the spatial trajectory traversed by the rat before the current location) to a single CA1 cell that codes for the current location (Bittner et al., 2017). Note that at the typical running speed of rodents, 50 cm correspond to several seconds of running. The broad activity of CA1 cells has therefore been interpreted as a predictive representation of upcoming events or places (Bittner et al., 2017). What could such an upcoming event be? For a rodent exploring a T-maze it might for example be important to develop a more precise spatial representation at the T-junction than inside one of the long corridors. With a broad CA1 place field located at the T-junction, information about the upcoming bifurcation could become available several seconds before the animal reaches the junction.

Bittner et al. interpreted their findings as the signature of an unusual form of STDP with a particularly long coincidence window on the behavioral time scale (Bittner et al., 2017). Given that the time span of several seconds between presynaptic stimulation and postsynaptic complex spike is outside the range of a potential causal relation between input and output, they classified the plasticity rule as non-Hebbian because the presynaptic neurons do not participate in firing the postsynaptic one (Bittner et al., 2017). As an alternative view, we propose to classify the findings of Bittner et al. as the signature of an eligibility trace that was left by the joint occurrence of a presynaptic spike arriving from CA3 (presynaptic factor) and a subthreshold depolarization at the location of the synapse in the postsynaptic CA1 neuron (postsynaptic factor); cf. Figure 2D. In this view, the setting of the synaptic flag is caused by a “Hebbian”-type induction, except that on the postsynaptic side there are no spikes but just depolarization, consistent with the role of depolarization as a postsynaptic factor (Artola and Singer, 1993; Ngezahayo et al., 2000; Sjöström et al., 2001; Clopath et al., 2010). In this view, the findings of Bittner et al. suggest that the synaptic flag set by the induction protocol leaves an eligibility trace which decays over 2 s. If a plateau potential (related to the third factor) is generated during these 2 s, the eligibility trace caused by the induction protocol is transformed into a measurable change of the synaptic weight. The third factor *M*^3*rd*^(*t*) in Equation (2) could correspond to the complex spike, filtered with a time constant of about 1 s. Importantly, plateau potentials can be considered as neuron-wide signals (Bittner et al., 2015) triggered by surprising, novel or rewarding events (Bittner et al., 2017). In this view, the results of Bittner et al. are consistent with the framework of neoHebbian three-factor learning rules. If the plateau potentials are indeed linked to surprising events, the three-factor rule framework predicts that *in vivo* many neurons in CA1 receive such a third input as a broadcast-like signal. However, only those neurons that also get, at the same time, sufficiently strong input from CA3 might develop the visible plateau potential (Bittner et al., 2015).

The main difference between the two alternative views is that, in the model discussed in Bittner et al. (2017), *each activated synapse* is marked by an eligibility trace (which is independent of the state of the postsynaptic neuron) whereas in the view of the three-factor rule, the eligibility trace is set only if the presynaptic activation coincides with a strong depolarization of the postsynaptic membrane. Thus, in the model of Bittner et al. the eligibility trace is set by the presynaptic factor alone whereas in the three-factor rule description it is set by the combination of pre- and postsynaptic factors. The two models can be distinguished in future experiments where either the postsynaptic voltage is controlled during presynaptic stimulation or where the number of simultaneously stimulated input fibers is minimized. The prediction of the three-factor rule is that spike arrival at a single synapse, or spike arrival in conjunction with a very small depolarization of <2 mV above rest, is not sufficient to set an eligibility trace. Therefore, LTP will not occur in these cases even if a calcium plateau potential occurs 1 s later.

## 4. Discussion and conclusion

### 4.1. Policy gradient vs. TD-learning

Algorithmic models of TD-learning with discrete states and in discrete time do not need eligibility traces that extend beyond one time step (Sutton and Barto, 1998). In a scenario where the only reward is given in a target state that is several action steps away from the initial state, reward information shifts, over multiple trials, from the target state backwards, even if the one-step eligibility trace connects only one state to the next (Sutton and Barto, 1998). Nevertheless, extended eligibility traces across multiple time steps are considered convenient heuristic tools to speed up learning in temporal difference algorithms such as *TD*(λ) or SARSA(λ) (Singh and Sutton, 1996; Sutton and Barto, 1998).

In policy gradient methods (Williams, 1992) as well as in continuous space-time TD-learning (Doya, 2000b; Frémaux et al., 2013) eligibility traces appear naturally in the formulation of the problem of reward maximization. Importantly, a large class of TD-learning and policy gradient methods can be formulated as three-factor rules for spiking neurons where the third factor is defined as reward minus expected reward (Frémaux and Gerstner, 2016). In policy gradient methods and related three-factor rules, expected reward is calculated as a running average of the reward (Frémaux et al., 2010) or fixed to zero by choice of reward schedule (Florian, 2007; Legenstein et al., 2008). In TD-learning the expected reward in a given time step is defined as the difference of the value of the current state and that of the next state (Sutton and Barto, 1998). In the most recent large-scale applications of reinforcement learning the expected immediate reward in policy gradient is calculated by a TD-algorithm for state-dependent value estimation (Greensmith et al., 2004; Mnih et al., 2016). An excellent modern summary of Reinforcement Learning Algorithms and their historical predecessors can be found in (Sutton and Barto, 2018).

### 4.2. Supervised learning vs. reinforcement learning

The experiments in Bittner et al. (2015, 2017) provide convincing evidence that plateau potentials are relevant for the described plasticity events and could be related to the third factor in three-factor rules. But in view of the difference between Equations (2) and (4) the question arises whether the third factor in the Bittner et al. experiments should be considered as a global or as a neuron-specific factor. Obviously, a plateau potential is neuron-specific. The more precise reformulation of this question therefore is whether this specificity is covered by a Type 2 factor written as $h_{i}\left(M^{3rd}\right)$ (see Equation 3) or whether it needs the more general Type 3 formulation with $M_{i}^{3rd}$ (see Equation 4). We see two potential interpretations.

- A surprise- or novelty-related global (scalar) neuromodulator *M*^3*rd*^ is capable of pushing all CA1 neurons into a state ready to generate a plateau potential, but only a fraction of the neurons actually receive this message and stochastically generate a plateau potential. The term $h_{i}\left(M^{3rd}\right)$ expresses the heterogeneity of this process. However, amongst the subset of neurons with $h_{i}\left(M^{3rd}\right)>0$ only those neurons that have a nonzero eligibility trace will implement synaptic plasticity. Thus the third factor is initially global, but triggers in the end very specific plasticity events limited to a few neurons and synapses only.
- A (potentially high-dimensional) mismatch-related error signal is randomly projected onto different neurons, including those in CA1. The effect is a neuron-specific third factor $M_{i}^{3rd}$ with index i. This second possibility is particularly intriguing because it relates to theories of attention-gated learning (Roelfsema and Holtmaat, 2018) and learning with segregated synapses (Guerguiev et al., 2017) as instantiations of approximate backpropagation of errors. The high-dimensional signal could be related to the mismatch between what the animal expects to see and what it actually sees in the next instant. In this second interpretation, we leave the field of generalized reinforcement learning and the experiments in Bittner et al. (2015, 2017) can be seen as a manifestation of supervised learning.

### 4.3. Specificity

If phasic scalar neuromodulator signals are broadcasted over large areas of the brain, the question arises whether synaptic plasticity can still be selective. In the framework of three-factor rules, specificity is inherited from the synaptic flags which are set by the combination of presynaptic spike arrival *and* an elevated postsynaptic voltage at the location of the synapses. The requirement is met only for a small subset of synapses, because presynaptic activity alone or postsynaptic activity alone are not sufficient; cf. Figure 1B. Furthermore, among all the flagged synapses only those that show, over many trials, a correlation with the reward signal will be consistently reinforced (Loewenstein and Seung, 2006; Legenstein et al., 2008).

Specificity can further be enhanced by an attentional feedback mechanism (Roelfsema and van Ooyen, 2005; Roelfsema et al., 2010) that restricts the number of eligible synapses to the “interesting” ones, likely to be involved in the task. Such an attentional gating signal acts as an additional factor and turns the three-factor into a four-factor learning rule (Rombouts et al., 2015; Roelfsema and Holtmaat, 2018). Additional specificity can also arise from the fact that not all neurons react in the same way to a modulator as implemented by the notation $h_{i}\left(M^{3rd}\right)$ of Type 2 rules (Brea et al., 2013; Rezende and Gerstner, 2014); as well as from additional factors that indicate whether a specific neuron in a population spikes in agreement with the majority of that population (Urbanczik and Senn, 2009). Maximal specificity is achieved with a neuron-specific third factor $M_{i}^{3rd}$ (Type 3 rules) as in modern implementations of supervised learning (Lillicrap et al., 2016; Guerguiev et al., 2017).

### 4.4. Mapping to neuromodulators

A global third factor is likely to be related to neuromodulators, but from the perspective of a theoretician there is no need to assign one neuromodulator to surprise and another one to reward. Indeed, the theoretical framework also works if each neuromodulator codes for a different combination of variables such as surprise, novelty or reward, just as we can use different coordinate systems to describe the same physical system (Frémaux and Gerstner, 2016). Thus, whether dopamine is purely reward related or also novelty related (Ljunberg et al., 1992; Schultz, 1998; Redgrave and Gurney, 2006) is not critical for the development of three-factor learning rules as long as dimensions relating to novelty, surprise, and reward are all covered by the set of neuromodulators.

Complexity in biology is increased by the fact that dopamine neurons projecting from the VTA to the striatum can have separate circuits and functions changing from reward in ventral striatum to novelty in the the tail of striatum (Menegas et al., 2017). Similarly, dopaminergic fibers starting in the VTA can have a different function than those starting in Locus Coeruleus (Takeuchi et al., 2016). Furthermore, findings over the last decade indicate that midbrain dopamine neurons generally show a high diversity of responses and input-output mappings (Fiorillo et al., 2013; Roeper, 2013). Finally, the time scale of eligibility traces could vary from one brain area to the next, in line with the general idea that higher cortical areas show more persistent activity than primary sensory areas (Wang and Kennedy, 2016). If the time scale of eligibility traces is slower in higher areas, we speculate that temporal links between more abstract, slowly evolving concepts could be picked up by plasticity rules. The framework of three-factor rules is general enough to allow for these, and many other, variations.

### 4.5. Alternatives to eligibility traces for bridging the gap between the behavioral and neuronal time scales

From a theoretical point of view, there is nothing—apart from conceptual elegance—to favor eligibility traces over alternative neuronal mechanisms to associate events that are separated by a second or more. For example, memory traces hidden in the rich firing activity patterns of a recurrent network (Maass et al., 2002; Jaeger and Haas, 2004; Buonomano and Maass, 2009; Susillo and Abbott, 2009) or short-term synaptic plasticity in recurrent networks (Mongillo et al., 2008) could be involved in learning behavioral tasks with delayed feedback. In some models, neuronal, rather than synaptic, activity traces have been involved in learning a delayed paired-associate task (Brea et al., 2016) and a combination of synaptic eligibity traces with prolonged single-neuron activity has been used for learning on behavioral time scales (Rombouts et al., 2015). The empirical studies reviewed here support the idea that the brain makes use of the elegant solution with synaptic eligibility traces and three-factor learning rules, but do not exclude that other mechanisms work in parallel.

### 4.6. The paradoxical nature of predictions in computational neuroscience

If a neuroscientist thinks of a theoretical model, he often imagines a couple of assumptions at the beginning, a set of results derived from simulations or mathematical analysis, and ideally a few novel predictions–but is this the way modeling works? There are at least two types of predictions in computational neuroscience, detailed predictions and conceptual predictions. Well-known examples of detailed predictions have been generated from variants of multi-channel biophysical Hodgkin-Huxley type (Hodgkin and Huxley, 1952) models such as: “if channel X is blocked then we predict that …” where X is a channel with known dynamics and predictions include depolarization, hyperpolarization, action potential firing, action potential backpropagation or failure thereof. All of these are useful predictions readily translated to and tested in experiments.

Conceptual predictions derived from abstract conceptual models are potentially more interesting, but more difficult to formulate. Conceptual models develop ideas and form our thinking of how a specific neuronal system could work to solve a behavioral task such as working memory (Mongillo et al., 2008), action selection and decision making (Sutton and Barto, 1998), long-term stability of memories (Crick, 1984; Lisman, 1985; Fusi et al., 2005), memory formation and memory recall (Willshaw et al., 1969; Hopfield, 1982). Paradoxically these models often make no detailed predictions in the sense indicated above. Rather, in these and other conceptual theories, the most relevant model features are formulated as *assumptions* which may be considered, in a loose sense, as playing the role of *conceptual predictions*. To formulate it as a short slogan: Assumptions are predictions. Let us return to the conceptual framework of three-factor rules: the purification of rough ideas into the role of three factors is the important conceptual work - and part of the assumptions. Moreover, the specific choice of time constant in the range of 1 s for the eligibility trace has been formulated by theoreticians as one of the model assumptions, rather than as a prediction; cf. the footnotes in section “Examples and theoretical predictions.” Why is this the case?

Most theoreticians shy away from calling their conceptual modeling work a “prediction,” because there is no logical necessity that the brain must work the way they assume in their model–the brain could have found a less elegant, different, but nevertheless functional solution to the problem under consideration; see the examples in the previous subsection. What a good conceptual model in computational neuroscience shows is that there *exists* a (nice) solution that should ideally not be in obvious contradiction with too many known facts. Importantly, conceptual models necessarily rely on assumptions which in many cases have not (yet) been shown to be true. The response of referees to modeling work in experimental journals therefore often is: “but this has never been shown.” Indeed, some assumptions may look far-fetched or even in contradiction with known facts: for example, to come back to eligibility traces, experiments on synaptic tagging-and-capture have shown in the 1990s that the time scale of a synaptic flag is in the range of one *hour* (Frey and Morris, 1997; Reymann and Frey, 2007; Redondo and Morris, 2011; Lisman, 2017), whereas the theory of eligibility traces for action learning needs a synaptic flag on the time scale of one *second*. Did synaptic tagging results imply that three-factor rules for action learning were wrong, because they used the wrong time scale? Or, on the contrary, did these experimental results rather imply that a biological machinery for three-factor rules was indeed in place which could therefore, for other neuron types and brain areas, be used and re-tuned to a different time scale (Frémaux et al., 2013)?

As mentioned earlier, the concepts of eligibility traces and three-factor rules can be traced back to the 1960s, from models formulated in words (Crow, 1968), to firing rate models formulated in discrete time and discrete states (Klopf, 1972; Sutton and Barto, 1981, 1998; Barto et al., 1983; Barto, 1985; Williams, 1992; Schultz, 1998; Bartlett and Baxter, 1999), to models with spikes in a continuous state space and an explicit time scale for eligibility traces (Xie and Seung, 2004; Loewenstein and Seung, 2006; Florian, 2007; Izhikevich, 2007; Legenstein et al., 2008; Vasilaki et al., 2009; Frémaux et al., 2013). Despite the mismatch with the known time scale of synaptic tagging in hippocampus (and lack of experimental support in other brain areas), theoreticians persisted, polished their theories, talked at conferences about these models, until eventually the experimental techniques and the scientific interests of experimentalists were aligned to directly test the assumptions of these theories. In view of the long history of three-factor learning rules, the recent elegant experiments (Yagishita et al., 2014; Brzosko et al., 2015, 2017; He et al., 2015; Bittner et al., 2017) provide an instructive example of how conceptual theories can influence experimental neuroscience.

## Author contributions

All authors listed have made a substantial, direct and intellectual contribution to the work, and approved it for publication.

### Conflict of interest statement

The authors declare that the research was conducted in the absence of any commercial or financial relationships that could be construed as a potential conflict of interest.

## Footnotes

^1^“Learning …(with dopamine) on striatal synapses …requires hypothetical traces of synaptic activity that last until reinforcement occurs and makes those synapses eligible for modification …”

^2^“(The eligibility trace *c*) decays to *c* = 0 exponentially with the time constant τ_*c*_ = 1 s.”

^3^“The time scale of the eligibility trace is assumed in this article to be on the order of seconds.”

^4^“Candidate weight changes *e*_*ij*_ decay to zero with a time constant τ_*e*_ = 500ms. The candidate weight changes *e*_*ij*_ are known as the eligibility trace in reinforcement learning.”

^5^“The time scales of the eligibility traces we propose, (are) on the order of hundreds of milliseconds,.. Direct experimental evidence of eligibility traces still lacks, …”
